# Representativeness of an air quality monitoring station for PM_2.5_ and source apportionment over a small urban domain

**DOI:** 10.1016/j.apr.2019.10.004

**Published:** 2020-02

**Authors:** S. Yatkin, M. Gerboles, C.A. Belis, F. Karagulian, F. Lagler, M. Barbiere, A. Borowiak

**Affiliations:** European Commission-Joint Research Centre, Directorate for Energy, Transport and Climate, 21027, Ispra, Italy

**Keywords:** Representativeness, Spatial distribution, Geostatistical analysis, PM_2.5_, CMB

## Abstract

In this study, PM_2.5_ concentrations together with the water-soluble ionic compounds and total elements were simultaneously measured at 16 sites in the city of Varese located in Northern Italy within a domain of 2 × 2 km^2^. The center point of this domain consisted of an existing urban air quality monitoring station. The representativeness of the monitoring station for PM_2.5_ mass and chemical composition was estimated using a methodology relying on statistical/geostatistical analyses. Source apportionment by means of the Chemical Mass Balance (CMB) receptor model was also performed to evaluate the spatial distribution of source contribution throughout the domain. Very high soluble fraction was found for Cd, Sb, K and V, indicating the anthropogenic origin of those elements. The geostatistical analysis/mapping showed that the monitoring station captured the spatial variation of PM_2.5_ and most of the anthropogenic originated elements, e.g., As, Cd and V, whereas it did not captured the spatial patterns of the ones originated from both natural and anthropogenic sources, e.g., Na, Ni, Pb, K, Zn, Fe, Cr, and Ti. The CMB source contribution estimations in the monitoring station were at least 25% different from many sites of the domain for PM_2.5_. The significant spatial variation in concentrations and source contribution estimates showed that the monitoring station could not be considered representative for the air quality monitoring studies with exposure assessment and source apportionment purposes in Varese.

## Highlights

• A methodology is proposed to check the representativeness of PM2.5 monitoring station in a small urban domain.
• Geostatistical analysis was combined with spatial distribution and solubility.
• Monitoring station captured spatial variation of PM_2.5_ and anthropogenic elements but not mixed sourced ones.
• CMB source contribution estimations in monitoring station were at least 25% different from many sites.

## 1 Introduction

Monitoring/sampling of atmospheric pollutants in research and epidemiological studies is generally performed at a limited numbers of sites assumed to be representative in surrounding area. However, ambient air concentrations at monitoring stations may differ significantly from neighboring sites, particularly at urban sites affected by multiple sources. According to EU Directive (2008/50/EC), at least one fixed monitoring station shall be installed for every 250,000 inhabitants to measure the annual average of atmospheric particulate matter (PM) fine fraction (PM_2.5_). There is no criterion on the capturing spatial variability of those fixed stations in the Directive. The concept of representativeness of monitoring stations has not been unambiguously and quantitatively defined yet (Spangl et al., 2007, Martin et al., 2015; Kracht et al., 2017a), however, several researchers proposed methods to quantify spatial variation in different domains, e.g., metropolitan areas, cities and traffic sites. Santiago et al. (2013) implemented computational fluid dynamic (CFD) modelling to two traffic sites for gaseous pollutants measured by passive samplers, and assessed the representativeness of fixed monitoring stations in the domains of ~700 × 800 m^2^. They reported a strong heterogeneity of the gaseous pollutants in the studied domains, which was interpreted that fixed monitoring stations are not representative for the nearby area. They suggested to use CFD model requiring knowledge about land use, traffic load and meteorological factors to assess the gaseous pollutants in such domains. Kanaroglou et al. (2005) and Kumar (2009) proposed location-allocation model to capture spatial variability of air pollutants for intra-urban exposure assessment and to determine optimally located fixed monitoring stations using land use and population data. Land use regression (LUR) models establish equations incorporating land data including altitude, traffic, residential area etc., meteorological data, and concentrations of air pollutants measured simultaneously in a domain to estimate current and future spatial distribution of concentrations (Montagne et al., 2013; Beelen et al., 2013; Johnson et al., 2010). Measurements at fixed monitoring stations are usually utilized to verify LUR estimates. Such models incorporating domain features and measurements have a great potential to be implemented for spatial distribution of pollutants and representativeness of fixed monitoring stations, however, such high-spatially resolved measurements of air pollutants are generally only available for gaseous pollutants using low-cost passive samplers (Ott et al., 2008 implemented for PM_2.5-10_), and micro-sensors (Mead et al., 2013).

In addition to gaseous pollutants, several studies aimed at evaluating spatial variability of PM concentrations over small domains using several sampling and monitoring methods, e.g., passive sampler (Ott et al., 2008), personal sampler (Nerriere et al., 2005, Violante et al., 2006; Duvall et al., 2012), particle counters (Merbitz et al., 2012a,b), dichotomous sampler (Kim et al., 2005) and cascade impactors (Miller et al., 2010; Tunno et al., 2016), with scope of exposure assessment and source apportionment. The metrics used to assess spatial variability in these studies ranged from geometric standard deviation to coefficient of divergence (COD), Pearson correlation and geostatistical analysis. All these studies reported heterogeneous dispersion of PM and associated components over small urban domains, which were attributed to meteorological/topographical factors, e.g., dominant wind direction, speed and stability of weather. In addition to assessing spatial variability, few studies compared concentrations at temporary sampling/monitoring sites with ones at fixed monitoring stations. Merbitz et al. (2012b) studied PM_10_ concentrations at 9 mobile and 3 fixed stations in an urban 160 km^2^ domain for a 9 days-period, and reported spatially and temporally variable PM_10_ results compared to those at the fixed stations. Miller et al. (2010) deployed one out of 50 mobile sampling sites at a fixed station in a 30 × 30 km^2^ domain covering one Canadian and one USA neighboring cities, and measured 3 PM fractions and associated organics. They implemented variogram analysis and ordinary kriging to construct a set of interpolated maps with autocorrelation of neighboring sites. Heterogeneity in spatial distributions of PM_1_ was attributed to heterogeneously spread local combustion sources and secondary organic particles formed by photochemical reactions. The heterogeneity of the PM_1-2.5_ fraction was found to be lower than the one of PM_1_, which was attributed to the effect of long-range transported particles. All measured pollutants were found to be higher in the fixed station than in the other sites. Duvall et al. (2012) measured PM_2.5_ and composition at 6 sites in a highly industrialized city, and compared those to the ones from a fixed monitoring site. In this study, chemical mass balance receptor model (CMB) was applied to the data from 7 sites, and significant intra-urban variability in PM_2.5_ and source contribution estimates of industrial sources were reported. Tunno et al. (2016) studied spatial variation of PM_2.5_ and composition at a ~2.8 km^2^ urban domain, dominated by traffic sources, which included 2 fixed stations. The samples were collected during day time between 7 a.m. and 7 p.m. over 5 days on the same filters at 36 sites in the domain for winter and summer. Substantial differences in PM and composition across sites were reported for two sampling seasons, even though PM_2.5_ concentrations were measured to be very similar by two within-domain monitoring stations. Usually, the concentrations at the fixed station located in a public park were lower than those in the neighboring sites. The authors concluded that the traffic sources, particularly urban buses with frequent stops, caused the substantial spatial variation in the concentrations. All these studies revealed that PM and composition around fixed monitoring stations can be substantially different, even in a small domain.

A number of studies have focused on the impact of parameters as temporal and spatial variability of air pollutants and instrumental imprecision on epidemiologic results by analyzing time series and modeled data (Wade et al., 2006; Goldman et al., 2010). Such factors were assessed using methods relying on statistical/geostatistical analysis of long-term collocated measurements in domains usually much bigger than aforementioned short-term studies. Using the log-transformed data, Wade et al. (2006) and Goldman et al. (2010) proposed a method to determine autocorrelation between monitoring sites in a metropolitan domain (~100 km*100 km) with 17 sampling sites. A semi-variogram using the auto correlation weighted by the temporal variance was modeled to quantify nugget and partial sill, which were considered as measures of instrumental precision and spatial variability, respectively. In addition, the impact of these two components on the health risk ratios was estimated. The authors reported that the error due to spatial variability resulted in average risk ratio reductions of less than 16% for secondary pollutants and between 43% and 68% for primary pollutants with intermediate values for pollutants of mixed origin (such as PM_2.5_), whereas the one due to precision error is only 6% (Goldman et al., 2010). They concluded that quantifying the impact of spatial variability on health effect estimates improves the interpretation of the epidemiological results.

The objective of this study is to determine the spatial variation of daytime PM_2.5_ concentrations and composition, and to evaluate the representativeness of fixed monitoring station located in the center of a small urban domain (Varese, Italy). A methodology including statistical/geostatistical analysis of measurements was applied to estimate the representativeness of the monitoring station. The solubility of elements was utilized to assess their origin, either natural, anthropogenic or long-range transported. CMB was applied to evaluate the source contribution estimates. Finally, an insight is given about the spatial variability of concentrations, source contributions and source profiles in a small city domain.

## 2 Materials and methods

### 2.1 Study area, sampling and analysis

**Fig. 1:**
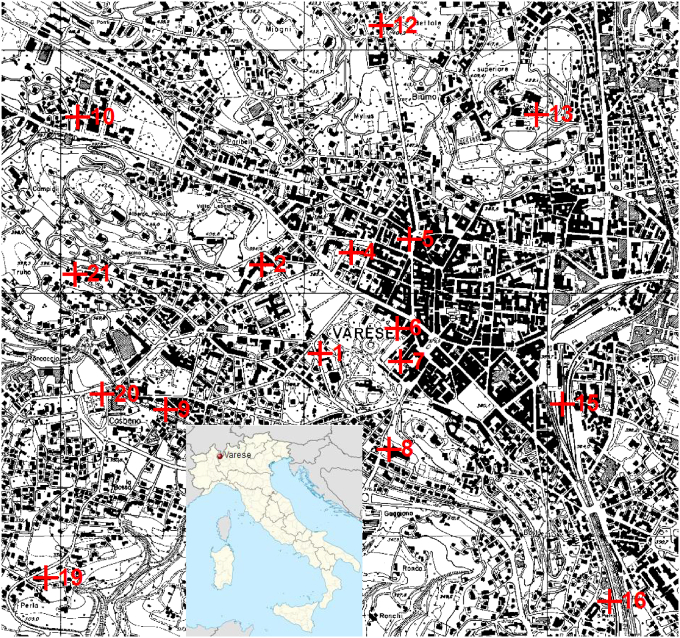
Location of Varese in Italy and map of the domain area with sampling sites shown with red crosses and site numbers. The fixed monitoring station is located in site #1.

The city of Varese (~82,000 inhabitants) lies at the feet of the Campo dei Fiori Mountain which creates a fence, direction west to east, at the north of city. The city also looks over the Lake Varese laying in the valley at the south of the city. Varese is built on the slope of the Campo dei Fiori Mountain with strong altitude increase from south to north. The rainfall is among the highest in Italy, with more than 1.500 mm of annual average. In winter, snow falls quite frequently, especially in January. Temperature typically varies from −1.5 °C to 27 °C and is rarely below -6 °C or above 31 °C. An urban traffic Air Quality Monitoring Station (AQMS) of the Regional Environment Agency (ARPA) is located in the city center surrounded by streets and train stations, and there are no important industrial sources nearby. The representativeness of AQMS was studied over a domain of 2 × 2 km^2^ by selecting 16 sampling sites (Fig. 1).

On 10th Feb 2010, daytime PM_2.5_ samples were simultaneously collected on PTFE filters (Pall Inc, Zefluor) using Derenda v3.1 (Comde-Derenda GmbH, Germany) Particulate Matter Low Volume Samplers (2.3 m³/hr). The weighing procedure, sample preparation, analytical methods and quality assurance/control can be found in the SI. It should be noted that the sample from site 5 was lost during the sample preparation, therefore, it was excluded from PM_2.5_ data analysis.

During sampling, at the AQMS, wind velocity was found to be 1.5 m/s in the morning, which then linearly decreased to 0.5 m/s in the evening. Temperature was 2.5 °C in the morning, reached a plateau of 6 °C in the noon, and decreased to 2.5 °C in the evening. Fog was present and relative humidity (RH) was inversely correlated with temperature changing between 70 and 50%. Meteorological factors, e.g., fog and RH, have a potential to impact the optical-based (not used in this study) and filter-based gravimetrical PM measurements due to water-intake by aerosols depending on hygroscopicity. All filters were conditioned prior to weighing in order to eliminate the influence of these factors on PM mass. In addition, RH spatial variation over the domain is expected to be small during sampling due to size of domain and orography, thus, RH is assessed to be not a factor affecting spatial variation of measured pollutants in this study.

### 2.2 Data analysis

Solubility was computed as the ratio of water-soluble concentrations to the total ones. Elements with high solubility were assumed to be of anthropogenic origin (Heal et al., 2005). Coefficient of divergence (COD, Eq. (1)) (Wongphatarakul et al., 1998) and relative standard deviation (RSD, Eq. (2)) were used to evaluate the homogeneity of concentrations over the domain (Ott et al., 2008). Generally, COD is used as a metrics of divergence between two sampling sites j and k over p time observations (Krudysz et al., 2009). COD values next to 0 and 1 respectively mean homogeneity (no difference) and absolute heterogeneity (max difference) between observations at two sampling sites. In the current study, COD of pollutant i, COD_i_, is computed using Eq. (1) for all data pairs. Additionally, Eq. (3) calculates COD_i,p_ to determine the contribution of site p to COD_i_:

(1) $$COD_{i}=\sqrt{\frac{1}{\frac{p\left(p-1\right)}{2}}\overset{p-1}{\underset{j=1}{\sum }}\overset{p}{\underset{k=j+1}{\sum }}\frac{{\left(C_{ij}-C_{ik}\right)}^{2}}{{\left(C_{ij}+C_{ik}\right)}^{2}}}$$

(2) $$RSD_{i}=\frac{\sqrt{\frac{1}{p-1}\sum_{j=1}^{p}{\left(C_{ij}-\bar{C_{i}}\right)}^{2}}}{\bar{C_{i}}}$$

(3) $$COD_{i,p}=\sqrt{\frac{1}{p-1}\overset{p}{\underset{k=1\;and\;k\neq p}{\sum }}\frac{{\left(C_{ip}-C_{ik}\right)}^{2}}{{\left(C_{ip}+C_{ik}\right)}^{2}}}$$

where, C_ij_, C_ik_ and C_ip_ are the concentrations of i at sampling sites j, k and p, and $\bar{C_{i}}$ is the average of i at all sampling sites.

Wilson et al. (2005) proposed a criterion of RSD>20% to indicate heterogeneity. US EPA proposed a criterion of COD>0.2 to indicate heterogeneity and a COD<0.1 to indicate homogeneity of concentration (USEPA, 2004). In this study, RSD and COD values lower than 0.2 were used to indicate homogenous distributions of concentrations.

Geostatistics is a branch of applied statistics that quantifies the spatial dependence and spatial structure of a measured property (Matheron, 1963). Commonly, geostatistical analysis includes two phases: spatial modelling called variography followed by spatial interpolation, the most common one being the kriging interpolation. Variography describes spatial correlation between observations according to the semivariance, γ(l), calculated using Eq. (4):

(4) γ(l)=1n(l)∑n=1n−1[z(Cy+l)−z(Cy)]2

where n(*l*) is number of sample pairs at a certain distance *l* (called lag or lag distance), and z(C_y_) and z(C_y+*l*_) are values of x, pollutant of interest, at locations y and y+*l*. Graphical representation of the semi-variance γ(*l*) versus lag distance is called experimental semivariogram or variogram. A variogram model is set by fitting a function to data pairs of lag distance and semi-variance. Linear, spherical or exponential models are the most commonly used, the spherical model (Eq. (5)) being generally the most successful one when spatial autocorrelation decreases to a point after which it becomes zero (Ott et al., 2008; Withworth et al., 2011; US-EPA, 2004).

(5) $$\left(\begin{array}{c} if\;l\leq a,\;\gamma =C_{0}+C_{1}\left[1.5l/a-0.5{\left(l/a\right)}^{3}\right]\\ if\;l>a,\;\gamma \left(l\right)=C_{0}+C_{1} \end{array}\right)$$

Spherical variogram shows a positive variance at intersection with y-axis, called “nugget” variance (Isaak and Srivastava, 1989). In general, from this point, semi variance increases until the maximum variance, called sill, is reached at “range” distance (Isaak and Srivastava, 1989). Range indicates distance from sampling points after which there is no more spatial correlation. Therefore, it is an indicator of area of representativeness for a fixed monitoring station.

Kracht et al. (2017b) proposed to apply point-centred variography in order to identify area of representativeness of an AQMS more accurately and precisely. In this method, point centred semivariance γ(l) is defined as the average of squared differences of within data pairs formed between AQMS and all other points in domain at each lag distance. Although being an accurate method, which reduces the required data pairs drastically, the limited number of available in this study makes it impossible to be applied. Therefore, PM_2.5_ concentrations and associated species were normalized to the values at AQMS (C/C_AQMS_) before mapping them to evaluate the area of AQMS representativeness. The relative expanded uncertainty of pollutant concentrations was estimated to be ~10% (see SI-1). Normalization to AQMS yields expanded uncertainty ~25%. Thus, the criterion of ±25% deviation from unity for C/C_AQMS_ was used to identify the representativeness. All variograms are fitted using the ordinary omni-directional kriging method. This methodology was applied for all measured species but only regulated pollutants in the European Air Quality Directive (2008/50/EC), namely PM_2.5_, Pb, Ni, As and Cd, are discussed further in this paper.

EPA-CMB was run to estimate the source contributions to PM_2.5_ in each sampling site. CMB calculates source contribution estimates (SCEs) to ambient concentrations using source chemical profiles and ambient measurements with their uncertainties. The details of CMB can be found elsewhere (EPA-CMB; SI-2). The source profiles were drawn from a previous study performed in the Lombardy Region, Northern Italy (Larsen et al., 2012), which are also available in the SPECIEUROPE repository (Pernigotti et al., 2016). Ammonium sulfate, ammonium nitrate (both secondary inorganic aerosol), wood burning, fuel-oil burning, natural gas burning, soil, cement industry and traffic were selected as possible sources for the CMB runs. The spatial distributions of CMB-SCEs to PM_2.5_ at the sampling sites were also evaluated using the abovementioned criterion of 25% difference from AQMS.

## 3 Results and discussion

### 3.1 Solubility, spatial variation and origin of pollutants

The results of the studied parameters and finding are reported in Table 1.

**Table 1:** Mean concentrations, RSD_i_, COD_i_, and solubility (mean±s). Range gives the presence of correlation between variance and distance. COD and RSD higher than 0.20 are shown in bold.

Species	Soluble Mean ngm^−3^	Variogram Model type	Nugget %	Range m	Sill %	Slope %/m	RSD_i_ %	COD_i_ %	Total Mean ngm^−3^	Variogram Model Type	Nugget %	Range m	Sill %	Slope %/m	RSD_i_ %	COD_i_ %	Solubility, mean±s %
PM_2.5_	n.a.	n.a.					n.a.	n.a.	36386	spherical	0.05	2500	3.5		17	14.5	n.a.
Cl^−^	84	nugget	6.1				**37**	**24**	n.a.	n.a.					n.a.		n.a.
NO_3_^−^	7075	linear	0			3.8 10^−3^	**31**	**21**	n.a.	n.a.					n.a.		n.a.
SO_4_^2−^	3168	linear	0			6.0 10^−4^	13	10	n.a.	n.a.					n.a.		n.a.
NH_4_^+^	3362	linear	0			5.0 10^−3^	**28**	19	n.a.	n.a.					n.a.		n.a.
Na	105	nugget	n.a.				**35**	**24**	228	nugget	8.9				**47**	**40**	51 ± 21
Mg	24	spherical	0.5	2500	3.7		15	12	36	spherical	0.05	2100	11.5		**26**	**21**	70 ± 17
Al	20	spherical	0.05	1500	4.5		20	13	68	nugget	16.7				**42**	**28**	33 ± 15
K	264	linear	0.5			5.5 10^−4^	18	13	313	linear	2.0			**7.3 10**^**−4**^	**22**	17	86 ± 6
Ca	68	nugget	5.3				**30**	**21**	126	nugget	11.2				**37**	**32**	59 ± 19
Ti	0.7	nugget	10.0				**74**	**42**	2.2	nugget	90.0				**76**	**46**	34 ± 17
V	0.5	spherical	0.18	1000	0.6		11	8	0.6	spherical	0.05	1000	0.5		14	**22**	85 ± 4
Cr	1.4	nugget	3.0				**21**	15	2.3	linear	0			**8.0 10**^**−4**^	**38**	**32**	65 ± 22
Mn	3.1	spherical	0.2	800	1.5		15	10	4.4	nugget	1.8				20	**22**	70 ± 7
Fe	59	nugget	5.0				**55**	**35**	130	nugget	4.3				**45**	**27**	46 ± 13
Co	0.04	spherical	1.0	1500	6.5		**31**	**27**	n.a.	n.a.					n.a.	na	n.a.
Ni	1.8	linear	0			0.21	**137**	**36**	5.2	spherical	1.2	1000	9		**58**	**35**	33 ± 19
Cu	4.7	nugget	4.5				**41**	**24**	7.6	nugget	n.a.				**37**	**24**	61 ± 8
Zn	32	linear	0			0.0013	10	7	45	nugget	9.1				**28**	**24**	76 ± 17
As	1.5	spherical	0.2	600	1.04		9	7	n.a.	n.a.					n.a.	n.a.	n.a.
Mo	1.6	nugget	2.5				**23**	16	3.1	spherical	2	1400	7		**30**	**20**	54 ± 9
Cd	0.27	spherical	0.01	1500	2.0		13	11	0.30	spherical		2100	2.4		13	12	88 ± 8
Sb	1.6	spherical	0.3	500	3.5		**26**	17	1.8	nugget	6.0				**28**	19	92 ± 5
Pb	8.0	linear	0			0.045	**69**	**33**	15	linear	0			**2.2 10**^**−3**^	**51**	**31**	54 ± 10

na.: Not available, **Linear**: the range could not be determined within the studied domain, **spherical**: the range is within the domain (<2800 m), **RSD**: relative standard deviation, **COD**: coefficient of divergence.

K, V, Cd and Sb were found to have a high water-soluble fraction (>80%) indicating their dominant sources being anthropogenic activities in the area. K, V, and Sb are the marker elements of biomass burning, fuel-oil and traffic (break wear), respectively (Belis et al., 2013). In the area, biomass and fuel-oil together with natural gas are used for residential heating purposes. Cd is likely arisen from long-range transportation. The solubility of Na, Mg, Ca, Cr, Mn, Cu, Zn, Mo and Pb were found to be between 50 and 80%, indicating contributions from natural and anthropogenic sources. Al, Ti, Fe and Ni are little water soluble, likely originating from natural sources. Ni is expected to be associated with V since they are both fingerprint elements of fuel-oil, however, in this study they were found to be not correlated.

**Fig. 2:**
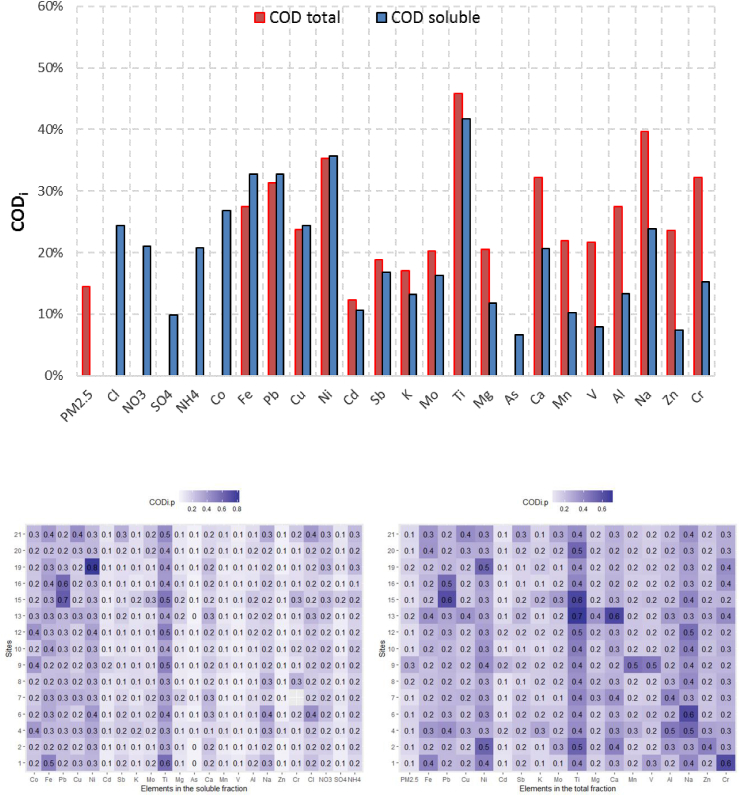
*Top*: coefficient of divergence (COD_i_) of each element in the total and soluble fractions; *bottom*: heat map contribution of each sampling site (#1 to #21) to the COD_i,p_ of soluble fraction (left) and total fraction (right).

Fig. 2 shows COD for all elements both in the total and soluble fractions. For the majority of elements, COD of soluble fractions (COD_sol_) were lower than the ones of the total fraction (COD_total_) with significant correlation (p < 0.01). Two different groups can be identified: *i)* COD_total_ is ~10–20% higher than COD_sol_ for Mg, As, Ca, Mn, V, Al, Na, Zn and Cr; and, *ii)* COD of two fractions are similar (relative difference < 10%) for Fe, Pb, Cu, Ni, Cd, Sb, K, Mo and Ti. COD_sol_ is generally lower than 20% with some exceptions (e.g., Co, Fe, Pb, Cu, Ni, Na, Cl, and NO_3_^−^) while COD_total_ is generally over 20% with a few exceptions (e.g., PM_2.5_, Cd, Sb, K, and As). The differences between COD_total_ and COD_sol_ were not found to be correlated with the mass ratio of soluble over total fractions.

Fig. 2 also shows two heat maps, which give the contribution of each sampling site to COD_i_s in two fractions. By visual checking of the heat maps, it is possible to evidence few vertical patterns rather than horizontal, showing that the majority of divergence comes from certain elements (e.g., Ni, and Ti) instead of sampling sites. There are no sampling sites that gives systematically high contribution to COD_i_s. Only COD_sol_ of site 19 for Ni is much higher (0.8) than the other sites. Moreover, this is the only COD_i,p_ of site 19 much higher than the rest of the soluble elements. Therefore, Ni concentration could be considered as an anomaly. High Ni content was found both in the soluble and total fraction and cannot be caused by an analytical mistake. In site 19, the sampler was installed in the garden of a private house along a road with medium traffic.

This part of the data analysis indicates that the study area as whole is affected by natural, anthropogenic and long-range transported sources with homogeneous distribution of species with high water solubility. In addition, few hot-spot areas were also observed for anthropogenic originated elements.

### 3.2 Variogram models

The variogram model types and nugget, range and sill values are also given in Table 1. “Spherical” indicates that a spherical model could be fitted in the variograms. Consequently, the range of spherical model remained within the domain. Overall, Table 1 showed that the maximum distance observed with spatial correlation between pollutants ranged between 500 and 2500 m. Conversely, “linear” indicates that the variance kept on increasing outside the study domain making it impossible to fit a spherical model. In this case, a linear model was fitted and the slope of the model is given in Table 1. It is likely to fit a spherical model with a more extended study domain, and the sill variance would have been reached outside the current study domain. Finally, the nugget indicates there is no spatial correlation. Three main patterns of variogram models were observed (see Fig. SI-1 for examples of each), and these were interpreted considering the associated COD_i_ and solubility:

1) Spherical fitting with homogenous distribution (RSD_i_ and/or COD_i_ < 0.2) for PM_2.5_, Mg, Al, V, Mn, As, and Sb in the soluble fraction, and V and Cd in the both fractions. This pattern suggested a correlation between variance and distance likely affected by local source(s) within the domain; however, the effect of source(s) was too small to produce heterogeneity. These elements had high water-soluble fractions indicating their anthropogenic origin. The water soluble parts of Al and Mn are likely deriving from local anthropogenic sources but not the insoluble fraction, which showed pure nugget pattern (#3 below).

2) Linear fitting with heterogeneous distribution (RSD_i_ and/or COD_i_ > 0.2) for NO_3_^−^, NH_4_^+^, and Ni, and Zn in the soluble fraction, Cr in the total fraction, and Pb in both. This pattern suggested that the effect of local source(s) was inhomogeneous over the domain.

3) Pure nugget with heterogeneous distribution for Na, Ca, Ti, Fe, and Cu for both fractions, Mo, Cr and Cl^−^ for soluble, and Al, Mn, Zn and Sb for total fraction. This pattern suggested two possible interpretations: several local sources contributing heterogeneously, or, a regional background contributing heterogeneously due to orography (likely in Varese) or microclimate (unlikely in Varese).

### 3.3 Spatial distribution of concentrations

**Fig. 3:**
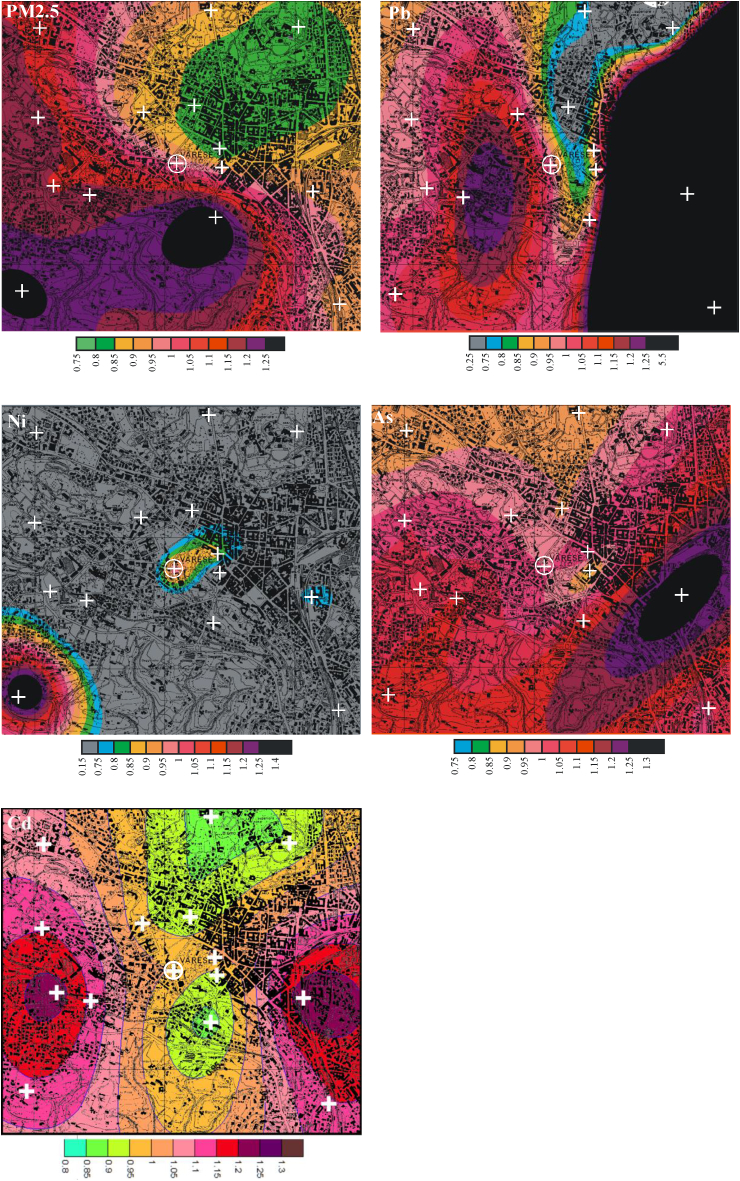
Contour maps of PM_2.5_, Pb, Ni, As-soluble and Cd concentrations normalized to AQMS. Crosses indicate the sampling sites (circled cross represents AQMS). Black and grey colors refer to values > 1.25 and < 0.75, respectively. Please note that the total of Site 5 is missing since the residual was lost during sample preparation.

The variograms were also used to plot krigged contour maps of the distribution C/C_AQMS_ values. The C/C_AQMS_ contour maps of PM_2.5_, Pb, Ni, Cd and As-soluble are illustrated Fig. 3. For PM_2.5_, the criterion of 25% was exceeded at the SW and S of AQMS. Aside from 2 sampling points at the S and SW, the distribution of PM_2.5_ can be considered to be homogenous, which is consistent with the COD_i_ and RSD_i_ values (Table 1). C/C_AQMS_ of PM_2.5_ increased from S-SW towards N, where the relative difference between the highest and lowest sampling points is about 50%. Similar pattern is also observed for the ionic species.

For a wide of area at the SE of AQMS, C/C_AQMS_ of Pb exceeded 125% while it was lower than 75% at the N. Similar pattern is observed for few homogenously distributed anthropogenic elements, e.g., Cd and As, and for heterogeneously distributed anthropogenic/natural elements, e.g., Cu, Zn and Fe. This makes the pattern difficult to interpret. In fact, the only important source at the E-SE of AQMS is the railway, which is expected to emit Fe, Mn, Cr and Cu, but, not Pb (Bukowiecki et al., 2007). This should be further investigated employing more sampling in the SE of AQMS.

For As, the criterion was exceeded at few sites at the SE of AQMS. For Ni, C/C_AQMS_ was lower than 75% nearly over the entire domain, while only at a site at the SW, it exceeded 125%. For Cd, the criterion was not exceeded but the map of Cd showed two hotspots in the W and E sites of the domain. The W site was characterized by the presence of two rail stations that could be somewhat associated the emission of Cd. For other pollutants, the criterion was generally exceeded, particularly for total concentrations. Conversely, the criterion was not exceeded for soluble fractions with few exceptions.

Overall, the concentration of PM_2.5_ showed a hot spot near AQMS and high concentration at the SW of the domain. Pb, As and Cd showed high concentrations at the E of the domain where rail station is located. On the other hand, Ni presented two main hotspots in the city center and at the SE of the domain. The significant concentration gradients (for Pb) and hot spots (for Ni, As ad Cd) suggest that, apart from long range transport, the distributions of these elements are likely affected by local sources within the studied domain.

### 3.4 Source contribution estimations by CMB

**Fig. 4:**
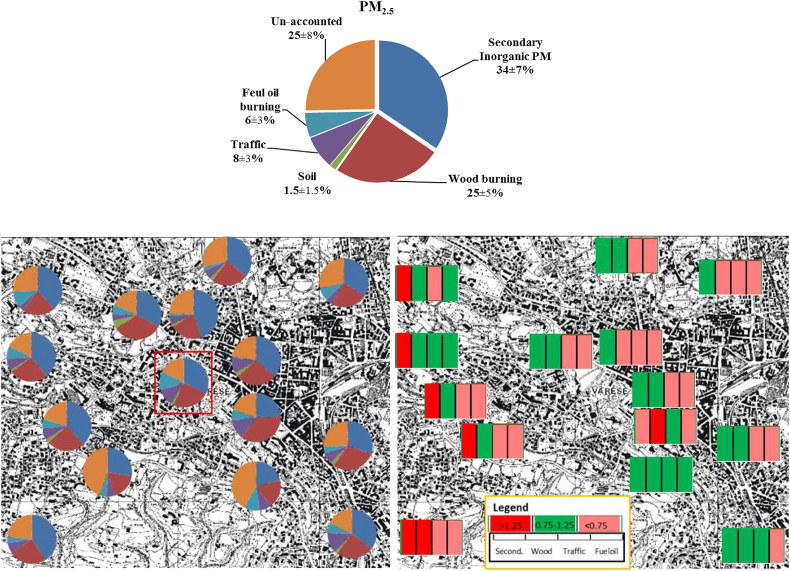
*Up*: Source contribution estimations (SCEs) by CMB (mean±s); *Bottom-left:* SCE by sampling site (red one is AQMS); *Bottom-right:* SCEs normalized to AQMS (SCE/AQMS). 1st, 2nd, 3rd and 4th bars represent SCE/AQMS of secondary inorganic PM, wood burning, traffic and fuel-oil, respectively. The dark red, light red and green colors of bars refer to SCE/AQMS >1.25, <0.75 and between 0.75 and 1.25, respectively.

The source contribution estimations (SCEs) of PM_2.5_ by CMB were plotted in Fig. 4 (mean±s). The CMB parameters, namely T-stat (>2), X^2^ (<1) and R^2^ (0.95–1.05) showed good performance of run. However, the ratio of estimated-to-measured (E/M) PM_2.5_ was generally below 80% (76 ± 6%, mean±s), while the relative uncertainty of estimated PM_2.5_ was calculated by CMB as 23 ± 2%, which is ~2.5 times higher than the input (10%). Thus, ~25% of measured PM mass could not be apportioned by CMB with the selected sources, which pointed another possible source(s). Many source profiles were derived from USEPA SPECIATE and SPECIEUROPE database for traffic, fuel-oil burning, soil and natural gas burning to obtain better CMB results than the above. However, these runs did not yield significantly better results than local sources used in this study. The lack of measured organic species is likely to result in underestimation of source contributions. The main sources contributed to PM_2.5_ were found to be secondary inorganic aerosols, wood burning, traffic, fuel-oil burning, and re-suspended soil.

For the 4 dominant contributors, namely secondary inorganic PM, wood burning, traffic and fuel-oil burning, the SCEs (in μg/cm^3^) normalized to AQMS (SCE/AQMS) were plotted in the bottom-right of Fig. 4. The SCE/AQMS of the secondary inorganic PM was found to be higher than 1.25 at the W of domain. The SCE/AQMS of wood burning remained between 0.75 and 1.25 throughout the domain. The SCE of traffic was found to be heterogeneously distributed, being one of the highest at the AQMS. Consequently, the SCE/AQMS of traffic was lower than 0.75 at many of the other sites, particularly at N and W. These parts of the city are less dense in population compared to vicinity of AQMS, thus, it is expected to have less traffic. The traffic density was not monitored during sampling, therefore, the relation between traffic density and SCE of traffic could not be assessed. Similar to traffic, the SCE of fuel-oil at AQMS was the highest, hence, the SCE/AQMS of this source was found to be lower than 0.75 at the remaining sampling sites. Due to lack of data on fuels used for residential heating purposes, the spatial variation of SCE/AQMS of fuel-oil could not be evaluated. Overall, the SCEs in the domain showed a difference of at least 25% between the sampling sites and AQMS, indicating that AQMS is not representative for the source contributions estimated by CMB using this data set for the city of Varese.

## 4 Conclusion

A methodology relying on geostatistical/statistical analysis of PM_2.5_ and elements/ions at 16 sampling sites over a small urban domain of 2  km × 2  km was applied to study the representativeness of a monitoring station. Geostatistical analysis was combined with spatial distribution of concentrations and solubility to evaluate the origin/sources of pollutants. A criterion of 25% difference in concentrations between sampling sites and the monitoring station was used to assess its area of representativeness. Using this methodology, the monitoring station was assessed to be representative for the whole domain for PM_2.5_ (with exception of two hotspot sites), Cd and As while it mainly overestimated Ni, and it could not capture the spatial variability of the very heterogeneously distributed Pb. It can be concluded that the monitoring station was suitable to capture the spatial variability of elements mainly originated from anthropogenic sources (e.g., As, Cd, and V), and was not suitable for elements generally originated from both natural and anthropogenic sources (e.g., Na, Ni, Pb, K, Zn, Fe, Cr, and Ti).

CMB source contribution estimations in the temporary sampling sites were at least 25% different than the monitoring station. For half of the sampling sites, the contributions from traffic and fuel-oil, the most variable contributors, were over 25% lower than at the monitoring station. On the other hand, in the west part of the domain, the contribution of secondary inorganic sources was found to be considerably higher than the monitoring station while the contribution of wood burning was more homogeneous over the domain.

One limitation of this study is that the one day-time series used to test this method was too short. To confirm the spatial variability we have found, longer measuring campaigns should be carried out. This methodology can also be applied to preliminary assessment at unmonitored area in order to determine the necessary number and location(s) of monitoring station(s).

Finally, the significant spatial variation of toxic pollutants and source contribution estimations for day-time data showed that the fixed station could not be considered representative for the air quality monitoring studies for exposure assessment and source apportionment purposes in Varese. Future Air Quality plans for Varese and any city should carefully evaluate target zones, which are potentially influenced by different sources, to capture spatial variability, and to set station(s) representative area for such purposes, ideally using more temporal data than one in this study.

## Declaration of competing interest

The authors whose names are listed immediately below certify that they have NO affiliations with or involvement in any organization or entity with any financial interest (such as honoraria; educational grants; participation in speakers’ bureaus; membership, employment, consultancies, stock ownership, or other equity interest; and expert testimony or patent-licensing arrangements), or non-financial interest (such as personal or professional relationships, affiliations, knowledge or beliefs) in the subject matter or materials discussed in this manuscript.
