# Supplementary material for: Representativeness of an air quality monitoring station for PM2.5 and source apportionment over a small urban domain
Source: Atmos Pollut Res. 2020 Feb;11(2):225–33. doi: 10.1016/j.apr.2019.10.004 (PMC6988503; doi:10.1016/j.apr.2019.10.004)
Supplement: Multimedia component 1 [file mmc1.docx]

**Supplementary Information for:**

**Representativeness of an Air Quality Monitoring Station for PM_2.5_ and Source Apportionment over a Small Urban Domain**

S. Yatkin*, M. Gerboles, C.A. Belis, F. Karagulian, F. Lagler, M. Barbiere and A. Borowiak

European Commission-Joint Research Centre, Directorate for Energy, Transport and Climate, 21027 Ispra, Italy

**SI 1. Analysis**

*Weighing*

Loaded and blank filters were conditioned for at least 2 days at 50% relative humidity and 20 ^o^C prior to weighing following the procedure of EN 14907 (2005). The filters were weighed using a microbalance with 1-μg resolution (Mettler Toledo, Switzerland, Model AX26). The balance was yearly calibrated using class-E1 mass blocks (Mettler Toledo, Switzerland), and drift checks with E2 mass block (Mettler Toledo, Switzerland) were performed prior to any weighing.

*IC*

The alcohol-wetted filters were placed into pre-cleaned 100 mL glass bottles. 25 ml of de-ionized water was added to each bottle. The filters were extracted by sonication in an ultrasonic bath for 30 min. Field, laboratory and reagent blanks (n=3 of each) were also extracted along with the samples. 5 mL of extract were filtered using Millex-HV, Syringe Driven filter (PVDF Durapore, 0.45 µm, Millipore, USA) prior to analysis. Cl^-^, NO_3_^-^, SO_4_^2-^ and NH_4_^+^ were analyzed using two IC Chromatographs (Dionex Cor., ICS-1000, USA) equipped with an autosampler (Dionex Cor., DV 1000, USA). Cl^−^, NO_3_^−^ and SO_4_^2−^ were determined using an anion-exchange column (IonPac AS14), carbonate/bicarbonate isocratic elution (flow of 0.38 ml min^−1^), an injection loop of 25 μL and an anion self-regenerating suppressor AMMS–2 mm with sulfuric acid. Na^+^, NH_4_^+^, K^+^, Mg^2+^ and Ca^2+^ were determined using a cation-exchange column (IonPac CS12A), methane sulfuric acid isocratic elution (flow of 0.25 ml min^−1^), an injection loop of 25 μL and a cation self-regenerating suppressor CMMS–2 mm with Tetrabutylammonium Hydroxide. Heated conductometric detectors were used for both chromatographs. The Chromeleon software was used to acquire and process the chromatograms.

The IC system was calibrated using certified mono-element standard solutions (Romil Ltd, UK, PriAg-xtra Series). Calibration curves were established analyzing freshly prepared calibration solutions, and they were accepted only if their correlation coefficients (r) were higher than 0.999. Linear calibration lines were plotted for anions/cations except for NH_4_^+^, for which a 2nd order polynomial was fitted through the calibration standard responses. Quality control (QC) solutions, prepared independently from calibration solutions, were analyzed just after the establishment of calibration curves, every 10th sample and at the end of a sequence. The instrument was re-calibrated if the drift exceeded 10%. When the drift laid between 3 and 10%, analytical results were corrected assuming a linear instrumental drift.

*ICP-MS*

10 mL of extracts were acidified to be pH<2 using high purity HNO_3_. The remaining filters and aliquots were digested together using Milestone Microwave Labstation Ethos 900 (Italy) following the procedure given in EN 14902 (2005) with modification of adding HF. Briefly, 8 mL ultrapure HNO_3_, 2 mL ultrapure H_2_O_2_ and 0.1 mL HF (Ultrex, JTBaker) were added into Teflon vessels and then placed in the Microwave. Field blanks, laboratory and reagent blanks (n=3 of each) and certified urban dust (NIST 1648, n=3) were digested and analyzed along the samples. The Teflon vessels were pre-cleaned by the same digestion program followed by 3 times rinsing with de-ionized water and drying in an oven at 150 °C, prior to usage. The sample containers (PTFE flasks) were soaked at least over night with 10% HNO_3_, followed by 3 times rinsing with the same acid, 3 times with de-ionized water and drying in an oven at 50 °C. The sample tubes of the autosampler were cleaned with the same procedure, but dried with filtered dry air.

An inductive coupled plasma-mass spectrometer (ICP-MS, Agilent Technologies Inc, Japan, 7500) was used to quantify Na, Ca, Al, K, Mg, Fe, Ti, Mn, Mo, Cu, Cr, Co, V, As, Ni, Cd, Sb, Pb and Zn in water and acid extracted samples. ICP-MS was calibrated using certified mono-element standard solutions (Romil Ltd, UK, PriAg-xtra Series). Calibration curves were established analyzing freshly prepared calibration solutions, and they were accepted only if correlation coefficients (r) were >0.999. The QC solution, prepared independently from calibration solutions, was analyzed just after the establishment of calibration curves, every 10th sample and at the end of a sequence. The instrument was re-calibrated if the drift exceeded 10%. The deviation during the analysis was corrected assuming the instrumental drift was linear between two QCs.

For the calculations of total elemental concentrations corresponding to the sum of the soluble and insoluble fractions, it was assumed that the water-soluble part does not contain water insoluble elements. The total concentrations were computed adding the water soluble elemental masses, which were calculated multiplying water-soluble concentrations in µg/L by 0.15 L (5 mL for IC+10 mL for ICP-MS), into the values found by analyzing remaining water soluble aliquots and filters.

The mean of laboratory blanks were subtracted from the sample concentrations. The method detection limits (MDLs) of IC and ICP-MS were calculated as three times standard deviation of laboratory blanks. The ionic concentrations were found to be higher than their MDLs. The mean sample-to-blank (S/B) ratios of Cl^-^, NO_3_^-^, SO_4_^2-^ and NH_4_^+^ were 4.3, 48.4, 236 and 47.0, respectively. The water-soluble elemental concentrations by ICP-MS were higher than their MDLs. The lowest of S/B ratios of water soluble elements ranged from 1.1 to 2.8 for Cr, Mg, Ca, Na, and Fe whereas was higher than 3 for the remaining elements. The lowest S/B ratios of total elemental concentrations ranged from 1.1 to 1.9 for Na, Mg, Al, K, Ca, Ti, as and Cd whereas the remaining were higher than 3. The total concentrations in all samples were found to be higher than their MDLs except Co and As, which were excluded from further data treatment.

Three certified dust (NIST 1648) samples were prepared implementing the same procedure: First, water soluble aliquots were extracted (no alcohol added), acidified and analyzed. Then, the remaining was digested and analyzed. The recovery of SRM 1648 remained between 80 and 120% except for Cr, (43±11%), Ni (67±26%), and Ti (77±7%).

*Measurement uncertainty*

The concentration of pollutant *i* of sample *j* (C_ij_) were calculated using Eq.1:

$C_{ij}=\frac{m_{ij}}{V_{air,j}}$ (1)

Where m_ij_ is the mass of pollutant *i* of sample *j* (µg) and V_air,j_ is the air volume (m^3^). PM_2.5_ mass was determined as the difference between post- and pre-weighing of filters. Mass of elements/ions was determined using Eq.2:

$m_{ij}=c_{ij}*v_{j}-c_{ib}*v_{b}$ (2)

Where c_ij_ and c_ib_ are the concentrations of samples and blanks by IC or ICP-MS, respectively; *v*_j_ and *v*_b_ are the volumes of samples and blanks, respectively. The measurement uncertainty was estimated following the Guide to the expression of uncertainty in measurement (JCGM, 2008). Any result of measurement, y, is a function of N parameters of x (Eq.3). Combined standard uncertainty, u_c_(y), is calculated using Eq.4:

$y=f(x_{1},x_{2},\ldots x_{N})$ (3)

$u_{c}^{2}(y)=\sum_{1}^{N} \left( \frac{\partial f}{\partial x} \right)^{2}u^{2}(x)$ (4)

The combined uncertainty of c_ij_, u_cij_, was estimated including contributions from ICP-MS reading (c_ij,0_) using external calibration curve, repeatability, recovery of Certified Reference Materials (CRM), volume, and standard uncertainty of single compound solutions. The uncertainty of ICP-MS reading, u_Cij,0_, was estimated using Eq.3 (EURACHEM / CITAC Guide CG 4, 2012).

$u_{cij,0}=\frac{S_{i}}{b_{i}}\left( \sqrt{\frac{1}{d}+\frac{1}{n}+\frac{\left( c_{ij,0-}\bar{c_{i}} \right)^{2}}{\sum_{q=1}^{p} \left( c_{iq-}\bar{c_{i}} \right)^{2}}} \right)$ (3)

Where c_iq_ is the concentration of standard *q* calculated using calibration curve; b_i_ is the slope of calibration curve; S_i_ is the residual standard deviation; *d* is the number of measurements used for calibration curves (d=6 standards*3 replicates); *n* is the number of replicates for each sample reading (n=3), and $\bar{c_{i}}$ is the mean concentration of standards. Repeatability was calculated as RSD/√n, RSD being the relative standard deviation of n replicates. Uncertainty of CRM-recovery was calculated as the standard deviation. Standard uncertainty of single compound solutions was found on their certificates. For standard and sample preparation, a 1 mL micropipette and series of volumetric flasks with 25 and 50 mL volume were used. The added volumes were checked by calibrated microbalance with 10 µg resolution. The uncertainty equation given in the calibration certification of balance was used to calculate the uncertainty of tare and final mass readings. The density of each added liquid given by manufacturers was used to convert mass to volume. Thus, the combined uncertainty of m_ij_ was calculated by applying Eq.4 to Eq.2.

The relative expanded uncertainty of C_ij_ was finally calculated using Eq.6, derived from applying Eq.4 to Eq.1:

$\frac{U_{Cij}}{C_{ij}}=k\sqrt{\left( \frac{u_{m_{ij}}}{m_{ij}} \right)^{2}+\left( \frac{u_{vair}}{V_{air,j}} \right)^{2}}$ (6)

The uncertainty of air volume and sampling was estimated to be 2% (Gerboles et al., 2011), and k was used as 2 for approximately 95% confidence level. For most of the pollutants, relative expanded uncertainty was estimated to be ~10%.

**SI 2. Performance Parameters of CMB model**

The performance of CMB run is controlled by four parameters: The T-statistics represents the ratio of the source contribution estimate to the standard error, and should be > 2.0. The coefficient of determination (R^2^) is described as the ratio of the source contribution estimate to the standard error, and should be > 0.8. χ^2^ is the weighed sum of squares of differences between estimated and measured fitting species concentrations and it should be < 4. The predicted/measured mass concentration should be between 80 and 120%.

**SI 3. Examples of Variogram Models**

Fig.SI-1 shows the examples of variogram models for “spherical” (PM_2.5_), ‘linear’ (NO_3_^-^) and “nugget” (Ti).

| 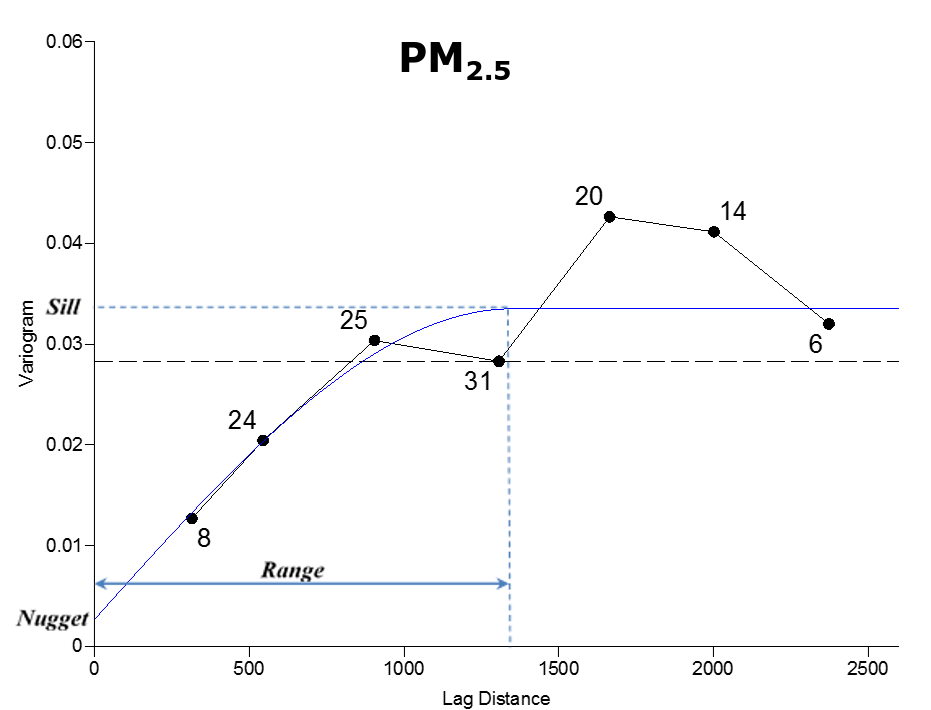 | 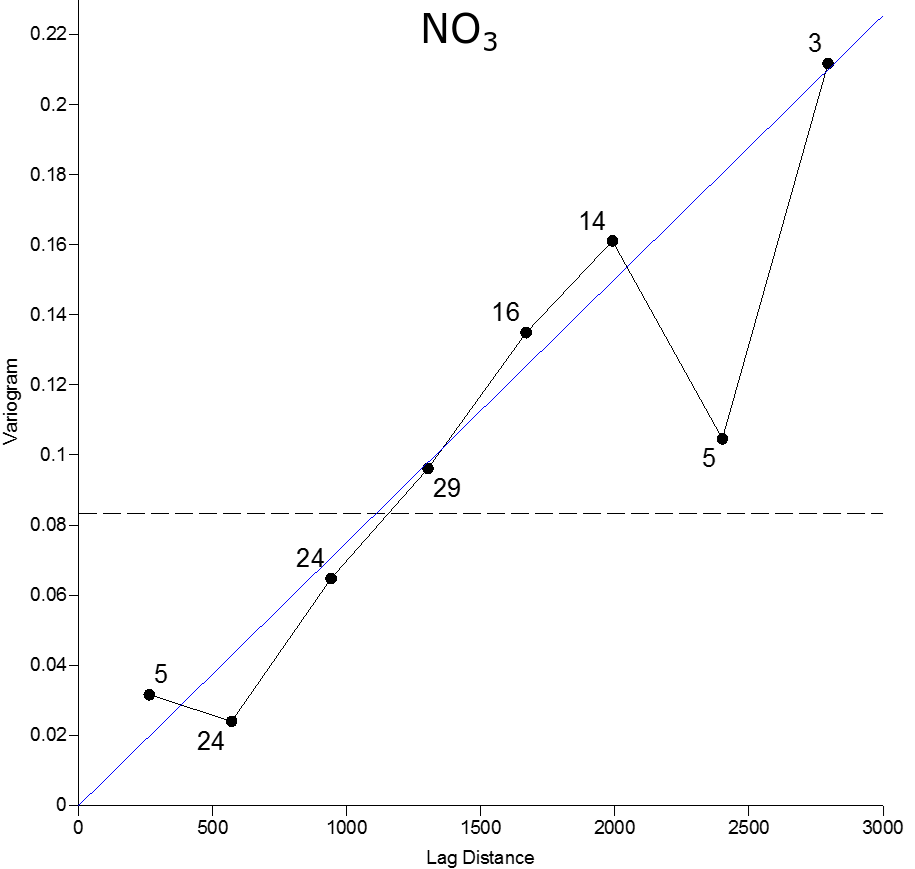 |
| --- | --- |
| 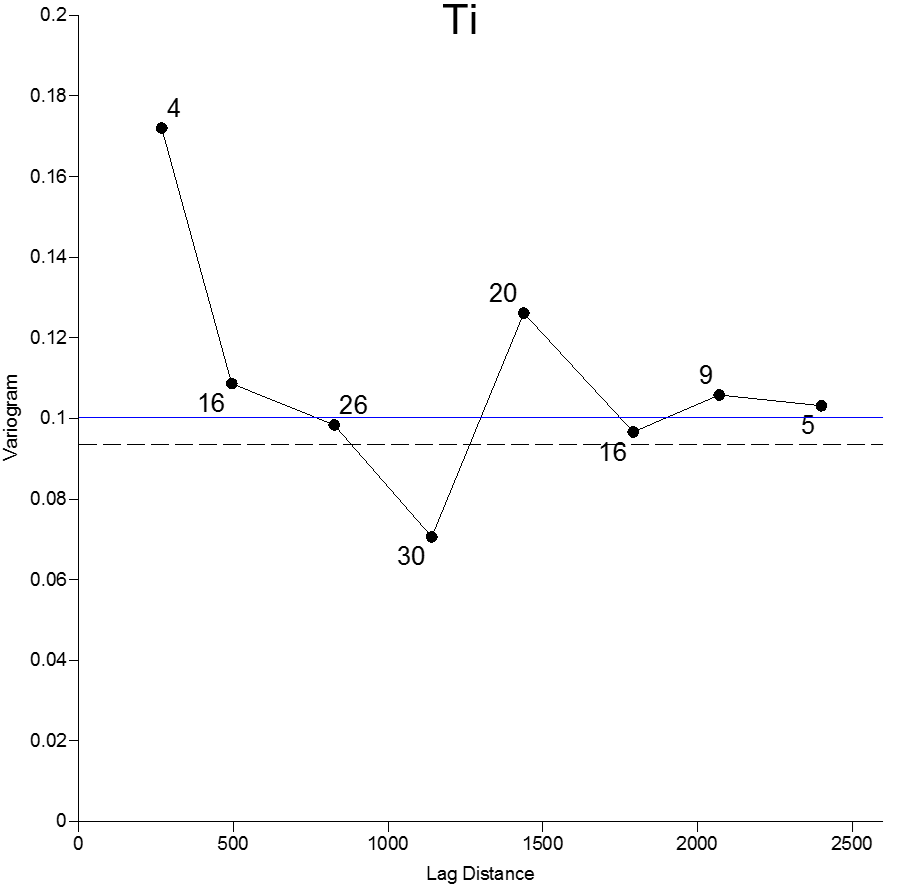 |  |

*Figure SI-1. Variogram for PM_2.5_, soluble NO_3_ and Ti mass concentrations. The labels over the variogram lags indicate how many pairs where used to compute the average semi-variance at each lag distance.*

**REFERENCES**

European Standard, 2005. Ambient air quality - Standard gravimetric measurement method for the determination of the PM2.5 mass fraction of suspended particulate matter. EN 14907. Brussels, Belgium. <http://www.CEN.eu>

EURACHEM / CITAC Guide CG 4, 2012. Quantifying Uncertainty in Analytical Measurement. 3^rd^ edition. Editors: S. L. R. Ellison and A. Williams. Available at <https://www.eurachem.org/images/stories/Guides/pdf/QUAM2012_P1.pdf>

Gerboles et al., 2011. Interlaboratory comparison exercise for the determination of As, Cd, Ni and Pb in PM_10_ in Europe. Atm.Env. 45(20), 3488-3499.

JCGM 100, 2008, Evaluation of measurement data — Guide to the expression of uncertainty in measurement. Available at <https://www.bipm.org/utils/common/documents/jcgm/JCGM_100_2008_E.pdf>
